# Genome-wide analysis of codon usage bias in Bovine Coronavirus

**DOI:** 10.1186/s12985-017-0780-y

**Published:** 2017-06-17

**Authors:** Matías Castells, Matías Victoria, Rodney Colina, Héctor Musto, Juan Cristina

**Affiliations:** 1 Laboratorio de Virología Molecular, Sede Salto, Centro Universitario Regional Litoral Norte, Universidad de la República, Gral. Rivera 1350, 50000 Salto, Uruguay; 2 Laboratorio de Organización y Evolución del Genoma, Unidad de Genómica Evolutiva, Instituto de Biología, Facultad de Ciencias, Universidad de la República, Iguá 4225, 11400 Montevideo, Uruguay; 3 Laboratorio de Virología Molecular, Centro de Investigaciones Nucleares, Facultad de Ciencias, Universidad de la República, Iguá 4225, 11400 Montevideo, Uruguay

**Keywords:** Bovine, Coronavirus, Codon usage, Evolution

## Abstract

**Background:**

Bovine coronavirus (BCoV) belong to the genus *Betacoronavirus* of the family *Coronaviridae.* BCoV are widespread around the world and cause enteric or respiratory infections among cattle, leading to important economic losses to the beef and dairy industry worldwide. To study the relation of codon usage among viruses and their hosts is essential to understand host-pathogen interaction, evasion from host’s immune system and evolution.

**Methods:**

We performed a comprehensive analysis of codon usage and composition of BCoV.

**Results:**

The global codon usage among BCoV strains is similar. Significant differences of codon preferences in BCoV genes in relation to codon usage of *Bos taurus* host genes were found. Most of the highly frequent codons are U-ending. G + C compositional constraint and dinucleotide composition also plays a role in the overall pattern of BCoV codon usage.

**Conclusions:**

The results of these studies revealed that mutational bias is a leading force shaping codon usage in this virus. Additionally, relative dinucleotide frequencies, geographical distribution, and evolutionary processes also influenced the codon usage pattern.

**Electronic supplementary material:**

The online version of this article (doi:10.1186/s12985-017-0780-y) contains supplementary material, which is available to authorized users.

## Background

Coronaviruses belong to the family *Coronaviridae* and are the largest enveloped single-strand RNA viruses, ranging from 26 to 31 kilobases in genome size [1, 2]. These viruses infect a wide range of avian and mammalian species, and are responsible for enteric or respiratory infections [3]. There is a rising concern about the emergence of two human coronaviruses, Severe acute respiratory syndrome-related coronavirus (SARS-CoV) and Middle-East respiratory syndrome coronavirus (MERS-CoV), who emerged in 2002 and 2012, respectively [4, 5]. Both SARS-CoV and MERS-CoV have a zoonotic origin, revealing the importance of the control of coronaviruses associated with domestic animals in close contact with human populations [6].

Coronaviruses consists of four genera named *Alpha-, Beta-, Gamma- and Deltacoronavirus* based on phylogenetic distance of highly conserved domains. In turn, *Betacoronavirus* genus is divided into four clades, namely A to D. Bovine coronavirus (BCoV) belongs to the *Betacoronavirus* genus clade A [7, 8].

BCoV are widespread around the world and cause enteric or respiratory infections among cattle [9, 10]. These viruses are associated with different syndromes in cattle, ranging from neonatal calf diarrhea, winter dysentery in adult cattle, to respiratory infection in cattle of different age groups [11].

BCoV infection leads to important economic losses to the beef and dairy industry throughout the world, associated with decreased performance, morbidity, mortality, direct cost of treatment of sick animals, and long-term effects on health and productivity of surviving calves [10, 12].

BCoV is closely related to the Human coronavirus OC43 (HCoV-OC43), isolated in 1967 [13]. Recent studies revealed that HCoV-OC43 resulted from a zoonotic transmission from bovine to human [6].

The redundancy of the genetic code provides evolution with the opportunity to adjust the efficiency and accuracy of protein production, while preserving the same amino acid sequence [13]. The relation of codon usage among viruses and their hosts may affect viral fitness, evasion from host’s immune system and evolution [14–16]. Synonymous triplets are generally not used randomly, and the main forces that drive this bias from equal usage are natural selection and mutational biases [17, 18]. Therefore, the study of codon usage in viruses can reveal important information about virus evolution, regulation of gene expression and protein synthesis [19].

In the present study, we performed comprehensive analyses of codon usage and composition of BCoV strains and explored the possible leading evolutionary determinants of the biases found.

## Methods

### Sequences

Complete genome sequences for 15 BCoV strains were obtained from GenBank database (available at: http://www.ncbi.nlm.nih.gov). For strain names and accession numbers see Additional file 1. By concatenation of different genome ORF’s sequences, different datasets were constructed: one dataset includes the concatenation of nonstructural region ORFs (ORF1ab), a second one containing the structural region ORFs (hemagglutinin-esterase, spike, envelope, membrane, nucleocapsid) and a third including the concatenation of nonstructural and structural ORFs. For each strain the ORFs were aligned using the MUSCLE program [20]. The alignment of concatenation of nonstructural and structural ORFs is available in Additional file 2.

### Data analysis

Codon usage, dinucleotide frequencies, base composition, the relative synonymous codon usage (RSCU) [21], the effective number of codons (ENC) [22], aromaticity (Aromo) and hydrophathicity (Gravy) values were calculated using the program CodonW (available at http://sourceforge.net/projects/codonw). The total G + C genomic content and G + C content at first, second and third codon positions were calculated using the Codon W program and EMBOSS Cusp program (available at http://emboss.bioinformatics.nl/cgi-bin/emboss/cusp). The RSCU values of *Bos taurus* cells were obtained from Kazusa database (available at: http://www.kazusa.or.jp/codon/). Codon usage preferences in BCoV in relation to the codon usage of *Bos taurus* were established by means of the use of the codon adaptation index (CAI) [23]. CAI was calculated using the approach of Puigbo et al. [24]. This method permits to compare a given codon usage (BCoV) to a predefined reference set (*Bos taurus*). In order to show whether the BCoV genes are well adapted to the codon usage of the reference set, as measured by CAI, we constructed a dataset composed of 22 *Bos taurus* genes selected at random and obtained from ARSA at DNA Database of Japan (available at: http:/http://www.ddbj.nig.ac.jp/arsa). The RCSU values of those 22 *Bos taurus* genes were found to be similar to the RCSU values obtained from the Kazusa database (see Additional file 3). Moreover, a strong positive correlation are found among the RSCU of those genes and the RSCU obtained from Kazusa database by Spearmans’rank correlation test [25] (*r* = 0.956, *P* < 0.00001).

Statistically significant difference among CAI values was determined by applying a Wilcoxom & Mann-Whitney test [25]. To discern if the statistically significant differences in the CAI values arise from codon preferences, we used e-CAI [26] to calculate the expected value of CAI (eCAI) at the 95% confident interval. A Kolmogorov-Smirnov test for the expected CAI was also performed [26].

### Multivariate analysis

Correspondence analysis (COA) is a type of multivariate analysis that permits a geometrical representation of the sets of rows and columns in a dataset [27, 28]. Each ORF is represented as a 59-dimensional vector and each dimension corresponds to the RSCU value of each codon (excluding AUG, UGG and stop triplets). Major trends within a dataset can be established using measures of relative inertia and genes ordered according to their position along the different axes [29]. COA was performed on the RSCU values by means of the use of the CodonW program. Correlation analysis was performed using Spearman’s rank correlation analysis method [25].

### Phylogenetic analysis

In order to gain insight into the genetic variability and evolution of BCoV, a phylogenetic tree analysis was performed for all BCoV strains enrolled in these studies, using complete genome codes. Sequences were aligned using the MUSCLE program [20]. Once aligned, the FindModel program (available at: https://www.hiv.lanl.gov/content/sequence/findmodel/findmodel.html) was used to identify the optimal evolutionary model that best fitted our sequence dataset.

Akaike information criteria (AIC) and the log of the likelihood (logL) indicated that the GTR+ Γ model was the most accurate (AIC = 60,864.73, logL = −30,423.36). Using this model, maximum likelihood trees were constructed using software from the MEGA 6 program [30]. As a measure of the robustness of each node, we employed the bootstrap method (500 replicas).

## Results

### General codon usage pattern in BCoV

**Fig. 1:**
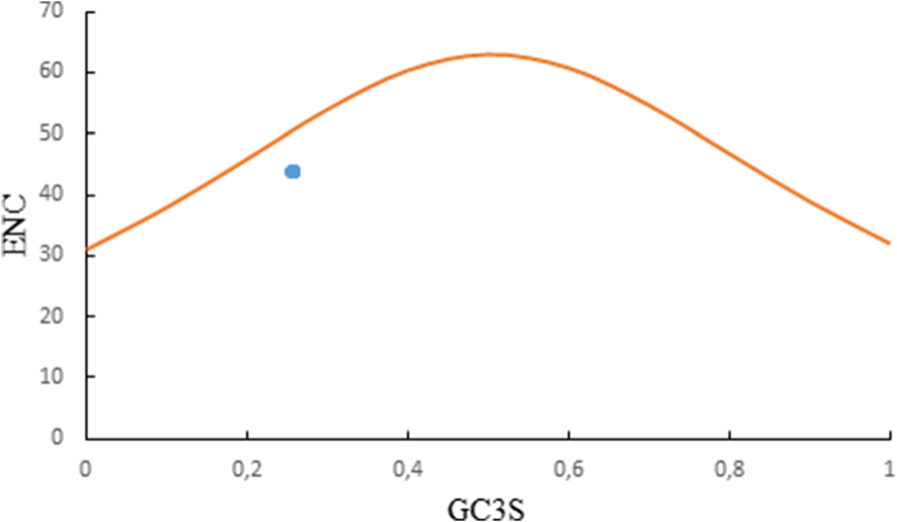
Effective number of codons (ENC) used in BCoV ORFs plotted against the GC3S The orange curve plots the relationship between GC3S and ENC in absence of selection. *Blue dots* show the results obtained for BCoV strains. Note that the values obtained for ENC and GC3S of all 15 BCoVs enrolled in these studies are very similar (SD of ±0.07 and ±0.0009, respectively) and for that reason they resemble a *single dot* in the figure

In order to gain insight into the degree of codon usage bias in BCoV, the ENC’s values were calculated for the complete genome of all BCoV strains. A mean value of 43.78 ± 0.07 was obtained for BCoV strains included in these studies. Then, a plot of ENC versus GC3S (ENC plotted against G + C content at the third codon position) was constructed. An ENC-GC3S plot of genes whose codon choice is constrained only by a GC3 mutational bias, will lie on or just below the continuous curve of the predicted ENC values [31]. As shown in Fig. 1, all points lie together under the expected ENC curve, indicating that G + C compositional constraints might play a role in ZIKV codon usage. Additionally, a correlation analysis between ENC and GC3S showed significant results (*r* = 0.811, *p* = 0.004).

The aromaticity (Aroma) and hydropathicity (Gravy) values of a given gene product can be indicative of the effect of translation or natural selection [32]. For these reasons, the effect of natural selection on BCoV codon usage was studied by correlation analysis between Gravy and Aroma values and nucleotide compositions at the third codon position and ENC values. No significant correlations between Gravy/Aroma values and nucleotide compositions at third codon position as well as between Gravy/Aroma and ENC values were observed (see Additional file 4).

These results indicate that codon bias in BCoV is related to mutational bias, whereas translational control, may be weak or absent in the reading frames of these viruses.

### Trends in codon usage variation across BCoV strains

To study the trends in codon usage variation among different BCoV genomes, we performed a COA analysis. COA was performed on the RSCU values for the ORF1ab of each BCoV strain enrolled in these studies and we examined the distribution of the strains in the plane defined by the first two principal axes of COA. The first axis generated by the analysis accounts for 43.37% of the total variation, while the second axis accounts for 18.96%. The results of these studies are shown in Additional file 5: Figure S1A. Interestingly, different BCoV isolates are located at different positions in the plane, suggesting that trends in variation of BCoV can be observed. Moreover, these trends correlate with the genetic variability observed by phylogenetic analysis (see Additional file 5: Figure S1B).

### Codon usage preferences in BCoV

To compare the codon usage preferences of BCoV with those of *Bos taurus*, the RSCU values of the codons in nonstructural, structural and complete BCoV genome ORFs were calculated and compared with those of *B. taurus*. The results of these studies are shown in Table 1.

**Table 1:** Codon usage in BCoV, displayed as RSCU^*a*^ values

AA	Cod	BT	NS	ST	Full	AA	Cod	BT	NS	ST	Full
Phe	*UUU*	0.84	1.78	1.68	1.76	Ser	*UCU*	1.03	2.00	1.92	1.98
	**UUC**	1.15	0.22	0.32	0.24		**UCC**	1.37	0.37	0.50	0.41
Leu	*UUA*	0.37	1.48	1.39	1.46		UCA	0.78	0.84	0.90	0.86
	*UUG*	0.71	2.09	1.62	1.98		UCG	0.39	0.14	0.26	0.18
	*CUU*	0.70	1.46	1.48	1.46	Pro	*CCU*	1.07	2.18	1.68	2.01
	**CUC**	1.25	0.22	0.47	0.28		**CCC**	1.39	0.38	0.82	0.53
	CUA	0.36	0.36	0.57	0.41		CCA	0.99	1.23	1.19	1.22
	CUG	2.58	0.39	0.48	0.42		CCG	0.53	0.21	0.31	0.24
Ile	*AUU*	0.98	1.78	1.66	1.74	Thr	*ACU*	0.88	1.91	2.21	2.00
	**AUC**	1.56	0.27	0.37	0.30		**ACC**	1.55	0.51	0.63	0.54
	AUA	0.45	0.95	0.97	0.96		ACA	1.00	1.32	0.93	1.20
Met	AUG	1.00	1.00	1.00	1.00		ACG	0.55	0.26	0.24	0.25
Val	*GUU*	0.64	2.24	2.20	2.23	Ala	*GCU*	1.00	2.12	2.07	2.11
	**GUC**	1.00	0.28	0.44	0.32		**GCC**	1.71	0.57	0.54	0.56
	GUA	0.39	0.63	0.75	0.65		GCA	0.80	1.13	1.12	1.13
	**GUG**	1.95	0.85	0.62	0.80		**GCG**	0.48	0.18	0.27	0.20
Tyr	*UAU*	0.78	1.63	1.57	1.61	Cys	*UGU*	0.83	1.55	1.54	1.55
	**UAC**	1.21	0.37	0.43	0.39		**UGC**	1.13	0.45	0.46	0.45
TER	UAA	**	**	**	**	TER	UGA	**	**	**	**
	UAG	**	**	**	**	Trp	UGG	1.00	1.00	1.00	1.00
His	*CAU*	0.75	1.56	1.56	1.56	Arg	*CGU*	0.48	2.17	1.56	2.01
	**CAC**	1.24	0.44	0.44	0.44		**CGC**	1.17	0.79	0.47	0.70
Gln	*CAA*	0.46	1.02	1.16	1.06		CGA	0.67	0.44	0.39	0.43
	**CAG**	1.53	0.98	0.84	0.94		**CGG**	1.32	0.32	0.27	0.31
Asn	*AAU*	0.81	1.65	1.70	1.67	Ser	*AGU*	0.87	2.09	1.71	1.98
	**AAC**	1.18	0.35	0.30	0.33		**AGC**	1.53	0.56	0.71	0.61
Lys	AAA	0.78	1.02	0.93	1.00	Arg	AGA	1.13	1.68	2.03	1.77
	AAG	1.21	0.98	1.07	1.00		AGG	1.20	0.59	1.29	0.78
Asp	*GAU*	0.84	1.70	1.40	1.64	Gly	*GGU*	0.63	2.50	2.46	2.49
	**GAC**	1.15	0.30	0.60	0.36		**GGC**	1.43	0.58	0.60	0.59
Glu	GAA	0.78	1.17	1.09	1.16		GGA	0.95	0.64	0.63	0.64
	GAG	1.21	0.83	0.91	0.84		GGG	0.98	0.28	0.30	0.28

^*a*^ *RSCU* relative synonymous codon usage, *AA* amino acid, *Cod* codons, *BT Bos taurus* cells, *NS* non structural genome region, *ST* structural genome region, *Full* complete genome coding regions. **, termination codons. Highly increased codons with respect to *B. taurus* cells (∆ ≥ 0.30) are shown in italics. Highly decreased codons with respect to *B. taurus* cells are shown in bold

The frequencies of codon usage in BCoV ORFs are significantly different in relation to *B. taurus* ones. Indeed, highly biased frequencies were found for UUU (Phe), UUA (Leu), UUG (Leu), CUU (Leu), AUU (Ile), GUU (Val), UAU (Tyr), CAU (His), CAA (Gln), AAU (Asn), GAU (Asp), UCU (Ser), CCU (Pro), ACU (Thr), GCU (Ala), UGU (Cys), CGU (Arg), AGU (Ser) and GGU (Gly). As can be seen, most of the highly preferred codons are U-ending and UpU containing codons, which strongly suggests that mutational bias is a main force shaping codon usage in BCoV (see Table 1). Moreover, most of the highly decreased codons frequencies with respect to *B. taurus* cells are C-ending codons, also suggesting a strong mutational bias in the use of these codons (Table 1). A Wilcoxom & Mann-Whitney test on the frequencies of BCoV codon usage among nonstructural and structural genome regions revealed no significant differences among both regions (*T* = 1983, *p*-value = 0.758).

### Codon usage adaptation in BCoV

In this study, a CAI metric was used as a measure of relative adaptedness of BCoV codon usage to *Bos taurus* host. CAI values for all triplets were calculated for the complete genome ORFs of BCoV strains enrolled in these studies, using *B. taurus* codon usage as the reference set. The results of these studies are shown in Table 2.

**Table 2:** Codon adaptation of BCoV genes in relation to *Bos taurus* codon usage, displayed as CAI^*a*^ values

	CAI-BT	%GC	%GC(1)	%GC(2)	%GC(3)
BCoV genes	0.638 ± 0.002	37.09 ± 0.000	45.86 ± 0.000	37.10 ± 0.000	28.32 ± 0.000
*Bos taurus* genes	0.756 ± 0.048	51.20 ± 6.803	52.17 ± 5.430	38.39 ± 5.546	63.04 ± 12.957

^*a*^ *CAI* codon adaptation index, *CAI-BT* codon adaptation index in relation to Bos taurus reference codon usage set. %GC, percentage of G + C genomic content, %GC(1) through (3), percentage of G + C genomic content at codon positions 1 through 3, respectively. In all cases, mean ± standard deviation values are shown

A mean value of 0.638 was obtained for BCoV genes in relation to *B. taurus*; while a mean CAI value of 0.756 was obtained for a *Bos taurus* sequence dataset in relation to the same reference set (see Table 2). In order to evaluate if the differences were statistically significant, we performed a Wilcoxon & Mann-Whitney test. The results of this test revealed that the differences in CAI values are statistically significant (*T* = 0, *p*-value <0.001). The CAI value obtained for *Bos taurus* genes is higher than the one obtained for BCoV in relation to *Bos taurus* codon usage (see Table 2). This reveals that BCoV genes are relatively less adapted to *Bos taurus* than *Bos taurus* genes itselves.

In order to discern if the statistically significant differences in CAI values arise from codon preferences [24], the expected CAI (e-CAI) values were calculated for BCoV complete genome ORFs sequences in relation to *B. taurus* codon usage reference set. The e-CAI algorithm [26] generated 500 random sequences with the same nucleotide and amino acid composition as the sequences of interest (in this case BCoV sequences). Then, we calculated the CAI values for all of them, and a Kolmogorov-Smirnov test for the e-CAI of these random sequences was performed in order to show if the generated sequences follow a normal distribution. The results of these studies revealed an e-CAI value of 0.656. Kolmogorov-Smirnov test revealed a normal distribution of the generated sequences (Kolmogorov-Smirnov test of e-CAI value of 0.028, which is below the critical value of 0.061). To avoid the effect of extreme compositional constraint and to make sure that CAI is directly correlated with codon usage preferences, Puigbo et al. [26] suggested that if the eCAI value of a gene is higher than its CAI value, it may be considered as evidence of codon usage adaptation. For all BCoV strains, CAI values were found to be lower than their corresponding eCAI values, when compared against *Bos taurus* (eCAI = 0.656, *p* < 0.05).

Taking all these results together, our studies revealed that the CAI values for BCoV genes are different from the CAI values obtained for *B. taurus* sequences and these differences are related to codon usage preferences.

It has been previously shown that dinucleotide biases can play a role in codon usage bias [29]. In order to determine if this is the case in BCoV, the relative abundances of the 16 dinucleotides in BCoV complete genome ORFs were established. The results of these studies are shown in Table 3.

**Table 3:** Relative abundance of dinucleotides in BCoV strains and summary of COA

		UU	UC	UA	UG	CU	CC	CA	CG
Mean (S.D^*.a*^)		2.02(±0.001)	0.60(<10^−3^)	1.44(=0)	1.61(<10^−3^)	0.92(<10^−3^)	0.44(±0.001)	0.82(±0.001)	0.25(<10^−3^)
Axis 1^*b*^	*r*	0.634615	0.163462	0.502747	0.574176	0.181319	−0.354396	0.717033	0.502747
	*P*	<0.05	0.568	0.080	<0.05	0.528	0.218	<0.05	0.080
		AU	AC	AA	AG	GU	GC	GA	GG
Mean (S.D.^*a*^)		1.47(=0)	0.70(=0)	1.24(<10^3^)	0.93(±0.001)	1.27(<10^−3^)	0.70(<10^−3^)	0.84(±0.001)	0.68(=0)
Axis 1^*b*^	*r*	0.502747	0.502747	0.502747	0.734890	0.431319	0.199176	0.288462	0.502747
	*P*	0.080	0.080	0.080	<0.05	0.133	0.490	0.317	0.080

^*a*^Mean values of BCoV strains relative dinucleotide ratios ± standard deviation. ^*b*^Correlation analysis between the first axis in COA and the sixteen dinucleotides frequencies in BCoV genes is shown

As can be seen, the relative abundance of UpU, UpA and ApU showed a strong deviation from the expected frequencies (i.e. 1.0) (a mean of 2.02, 1.44 and 1.47, respectively), while UpC, CpC, ApC and GpC frequencies were markedly underrepresented (a mean of 0.60, 0.44, 0.70 and 0.70, respectively). The relative abundance of CpG and GpC also showed a strong deviation from the expected frequencies (i.e. 1.0) (a mean of 0.25 and 0.70, respectively).

## Discussion

In these studies, we first study the general codon usage pattern in BCoV. When the ENC’s values were calculated for the complete genome of all BCoV strains, a mean value of 43.78 ± 0.07 was obtained. Since the ENC values obtained are >40, these results suggest a relatively conserved codon usage bias among different BCoV genomes. This is in agreement with previous reports in other members of the family, like SARS-CoV (mean ENC = 48.99) [33], the avian coronavirus Infectious bronchitis virus (ENC = 42.79) [34] or the Porcine epidemic diarrhea virus (ENC = 47.91) [35]. The ENC-GC3S plot revealed that all values obtained for BCoV lie bellow the continuous curve of predicted ENC values in absence of selection, revealing that G + C compositional constrain play a role in BCoV codon usage (Fig. 1).

Moreover, no significant correlations between both Gravy and Aroma values with nucleotide composition at the third codon position, as well as ENC values (see Additional file 3). This indicates that the role of translational selection in BCoV codon usage bias is weak or absent. Taking all together, the results of these studies suggests that mutational bias is a main force shaping codon usage in this virus.

Then, in order to study the trends in codon usage variation across BCoV strains, a COA analysis was performed on the RSCU values for the ORF1ab of each BCoV strain enrolled in these studies. Different BCoV isolates are located at different positions in the plane defined by the first two principal axes of COA, revealing that trends in codon usage variation can be observed (see Additional file 5: Figure S1A). This is also in agreement with the results found in the phylogenetic analysis of BCoV strains enrolled in these studies (see Additional file 5: Figure S1B) and with recent studies on genetic variability of coronaviruses, showing that BCoVs strains are distributed on three main sub-clusters named C1, C2, and C3 [6]. Sub-cluster C1 includes BCoVs from America and Asia, sub-cluster C2 includes BCoVs from Europe and sub-cluster C3 includes prototype, vaccine, or attenuated BCoV strains [6]. As can be seen in Additional file 5: Figure S1A, BCoV strains Mebus and Quebed, who belong to C3 cluster, are situated in different positions in the plane defined by the first two axes of COA in relation to C1 strains. Moreover, different genetic lineages of C1 sub-cluster are also located at different positions in the plane (Additional file 5: Figure S1A). This is in agreement with the results found in the phylogenetic analysis of BCoVs enrolled in these studies (Additional file 5: Figure S1B). Moreover, BCoV strain BCV-AKS-01, who shows a more distant genetic relation with C3 and C1 BCoVs enrolled in the studies (Additional file 5: Figure S1B), is situated in a different position in the plane defined by the first two axes of COA (Additional file 5: Figure S1A). The results of these studies revealed that evolutionary processes also influenced the codon usage pattern of BCoV.

In these studies, significant differences in codon usage frequencies were found among BCoV and *B. taurus* ones (Table 1). Interestingly, recent studies on human coronaviruses revealed significant biases in nucleotide composition [36]. While the A/G bias is a relatively stable property among coronaviruses, the C/U bias differs significantly in each virus type, with U-counts ranging from 30.7% (SARS-CoV) to 40.3% (HCoV-HKU) and C-counts from 20.3% (MERS-CoV) to 12.9% (HCoV-HKU) [36]. The U-count for BCoV revealed a value of 35.6%, while the C-count shows a value of 15.1%. This is in agreement with the results found in this work and may help to explain the bias found in BCoV genome composition and codon usage. Moreover, the CAI values for BCoV genes resulted to be significantly different from the ones obtained for *B. taurus*, revealing differences in codon usage preferences (Table 2).

The results of these studies revealed that the relative abundance of CpG and GpC dinucleotides showed a strong deviation from the expected frequencies (Table 3). The under-representation of CpG might be due to its immunostimulatory properties as recognition of unmethylated CpG by Toll like receptor 9 (TLR9), which leads to activation of several immune response pathways in the host [37]. Moreover, an increase in CpG dinucleotide frequency has been shown to lead to attenuation of replication in RNA viruses [38]. Cytosine deamination and selection against CpG motifs have been proposed as two independent selection forces that shape codon usage bias in coronaviruses [39], suggesting that immune selection may play a role in the observed BCoV codon usage bias. This is in agreement with the results of this work and indicates that the composition of dinucleotides also determines the variation in synonymous codon usage among BCoV.

## Conclusions

The results of these studies revealed significant differences in codon preferences in BCoV genes in relation to *B. taurus* codon usage. The overall codon usage among BCoV strains is similar. All U- ending codons are highly frequent codons, which strongly suggests that mutational bias is a leading force shaping codon usage in this virus. G + C compositional constraint influences the codon usage of BCoV. Dinucleotide composition also plays a role in the overall pattern of BCoV codon usage.

## Additional files

Additional file 1: Origins of the BCoV strains. A table showing strain names and accession numbers. (DOCX 11 kb)

Additional file 2: Alignment of concatenation of nonstructural and structural ORF’s of Bovine coronaviruses. A fasta file originated using the MUSCLE program [20]. (FAS 378 kb)

Additional file 3: Comparison of *Bos taurus* RSCU obtained using Kazusa databasea and the RSCU of *Bos taurus* genes dataset used in these studies. (DOCX 14 kb)

Additional file 4: The correlation between Gravy and Aroma and nucleotide at the third codon position of each codon and ENC values. (DOCX 12 kb)

Additional file 5: Figure S1. In (A) Positions of the BCoV strains in the plot of the first two major axes by correspondence analysis (COA) of relative synonymous codon usage (RSCU) values. In (B) Maximum likelihood phylogenetic analysis of BCoV complete codes. (DOCX 67 kb)

## Abbreviations

AIC: Akaike information criteria
Aroma: Aromaticity
*B. taurus*: *Bos taurus*
BCoV: Bovine Coronavirus
CAI: Codon adaptation index
COA: Correspondence analysis
eCAI: Expected value of CAI
ENC: Effective number of codons
Gravy: Hydrophathicity
GTR+ Γ: General Time Reversible plus gamma model
HCoV: Human Coronavirus
logL: The log of the likelihood
ORF: Open reading frame
RSCU: Relative synonymous codon usage
SARS-CoV: Severe Acute Respiratory Syndrome-related coronavirus (SARS-CoV) and Middle-East Respiratory Syndrome coronavirus (MERS-CoV)
