# Supplementary material for: Genome-wide analysis of codon usage bias in Bovine Coronavirus
Source: Virol J. 2017 Jun 17;14:115. doi: 10.1186/s12985-017-0780-y (PMC5474002; doi:10.1186/s12985-017-0780-y)
Supplement: Supplementary file 1 — Origins of the BCoV strains. A table showing strain names and accession numbers. (DOCX 11 kb) [file 12985_2017_780_MOESM1_ESM.docx]

**Additional file 1. Origins of the BCoV strains.**

**___________________________________________________________________**

**Strain Accession number**

___________________________________________________________________

BCV-AKS-01 KU886219

BCoV-LUN AF391542

BCoV-ENT AF391541

Bovine Coronavirus NC_003045

E-AH187 EF424619

E-AH187-TC FJ938064

R-AH187 EF424620

R-AH-65-TC EF424618

R-AH-65 EF424617

E-AH-65-TC EF424616

E-AH-65 EF424615

DB2 DQ811784

E-DB2-TC FJ938063

Mebus U00735

Quebec AF220295

_____________________________________________________________________
