# Supplementary material for: Genome-wide analysis of codon usage bias in Bovine Coronavirus
Source: Virol J. 2017 Jun 17;14:115. doi: 10.1186/s12985-017-0780-y (PMC5474002; doi:10.1186/s12985-017-0780-y)
Supplement: Supplementary file 3 — Comparison of Bos taurus RSCU obtained using Kazusa databasea and the RSCU of Bos taurus genes dataset used in these studies. (DOCX 14 kb) [file 12985_2017_780_MOESM3_ESM.docx]

**Additional file 3. Comparison of *Bos taurus* RSCU obtained using Kazusa database*^a^* and the RSCU of *Bos taurus* genes dataset used in these studies.**

**______________________________________________________________________**

AA*^b^* Codon Frequency*^c^*

______________________________________________________________________

Kazusa database *Bos taurus* genes dataset

____________________________________________________

Gly GGG 16.79 13.53

Gly GGA 16.15 13.53

Gly GGT 10.85 7.04

Gly GGC 24.39 23.39

Glu GAG 41.92 33.82

Glu GAA 26.92 26.07

Asp GAT 20.53 19.30

Asp GAC 28.16 25.37

Val GTG 30.84 31.29

Val GTA 6.30 5.35

Val GTT 10.08 11.83

Val GTC 15.89 16.77

Ala GCG 8.61 7.04

Ala GCA 14.32 17.47

Ala GCT 17.87 16.49

Ala GCC 30.51 26.49

Arg AGG 11.36 13.53

Arg AGA 10.74 8.31

Ser AGT 10.98 10.85

Ser AGC 19.31 19.02

Lys AAG 34.69 35.80

Lys AAA 22.37 26.49

Asn AAT 14.70 17.33

Asn AAC 21.38 26.49

Met ATG 22.47 27.34

Ile ATA 6.67 8.59

Ile ATT 14.59 12.40

Ile ATC 23.32 29.59

Thr ACG 7.20 6.48

Thr ACA 13.02 11.41

Thr ACT 11.48 12.40

Thr ACC 20.05 19.87

Trp TGG 13.49 14.94

End TGA 1.32 1.12

Cys TGT 9.30 12.82

Cys TGC 12.57 14.51

End TAG 0.63 0.56

End TAA 0.70 1.26

Tyr TAT 11.41 12.26

Tyr TAC 17.46 19.87

Leu TTG 12.03 16.20

Leu TTA 6.34 5.92

Phe TTT 16.38 19.45

Phe TTC 22.34 22.83

Ser TCG 4.97 4.36

Ser TCA 9.92 8.59

Ser TCT 13.13 15.36

Ser TCC 17.30 16.06

Arg CGG 12.49 9.30

Arg CGA 6.42 5.35

Arg CGT 4.64 2.67

Arg CGC 11.06 9.16

Gln CAG 35.03 32.69

Gln CAA 10.48 10.71

His CAT 9.38 11.13

His CAC 15.50 17.19

Leu CTG 43.51 44.25

Leu CTA 6.05 5.63

Leu CTT 11.86 15.08

Leu CTC 21.17 19.02

Pro CCG 7.80 7.32

Pro CCA 14.65 12.68

Pro CCT 15.81 14.23

Pro CCC 20.36 16.49

______________________________________________________________________

*^a^* Available at: http://www.kazusa.or.jp. *^b^* AA, Amino acid. *^c^* Frequency, frequency per thousand codons.
