# Supplementary material for: Genome-wide analysis of codon usage bias in Bovine Coronavirus
Source: Virol J. 2017 Jun 17;14:115. doi: 10.1186/s12985-017-0780-y (PMC5474002; doi:10.1186/s12985-017-0780-y)
Supplement: Supplementary file 4 — The correlation between Gravy and Aroma and nucleotide at the third codon position of each codon and ENC values. (DOCX 12 kb) [file 12985_2017_780_MOESM4_ESM.docx]

**Additional file 4. The correlation between Gravy and Aroma and nucleotide at the third codon position of each codon and ENC values.** ____________________________________________________________________________________________________________________

U3s C3s A3s G3s GC3s ENC

____________________________________________________________________________________________________________________

Gravy *r* 0.340 -0.339 0.069 -0.686 -0.635 -0.562

*P* 0.230 0.275 0.764 0.022 0.085 0.056

____________________________________________________________________________________________________________________

Aromo *r* -0.326 0.418 -0.009 -0.356 0.109 0.101

*P* 0.284 0.131 0.944 0.254 0.509 0.689

____________________________________________________________________________________________________________________
