# Supplementary material for: Genome-wide analysis of codon usage bias in Bovine Coronavirus
Source: Virol J. 2017 Jun 17;14:115. doi: 10.1186/s12985-017-0780-y (PMC5474002; doi:10.1186/s12985-017-0780-y)
Supplement: Supplementary file 5 — In (A) Positions of the BCoV strains in the plot of the first two major axes by correspondence analysis (COA) of relative synonymous codon usage (RSCU) values. In (B) Maximum likelihood phylogenetic analysis of BCoV complete codes. (DOCX 67 kb) [file 12985_2017_780_MOESM5_ESM.docx]

**(A)**

**(B)**

**BCoV/R-AH187**

**BCoV/E-AH187-TC**

**BCoV/LUN**

**BCoV/ENT**

**BCoV/NC 003045**

**BCoV/E-AH65**

**BCoV/E-AH65-TC**

**BCoV/R-AH65-TC**

**BCoV/R-AH65**

**BCoV/E-AH187**

**BCoV/DB2**

**BCoV/E-DB2-TC**

**BCoV/Mebus**

**BCoV/Quebec**

**BCoV/BCV-AKS-01**

**100**

**100**

**100**

**100**

**100**

**99**

**98**

**100**

**0.001**

**C3**

**C1**

**Additional file 5: Figure S1. In (A) Positions of the BCoV strains in the plot of the first two major axes by correspondence analysis (COA) of relative synonymous codon usage (RSCU) values.** The first and second axes account for 43.37 % and 18.96 % of the total variation, respectively. Strains belonging to BCoV sub-cluster C3 [6] are shown in red, strains belonging to different genetic lineages in sub-cluster C1 [6] are shown in green, violet and blue. Strain BCV-AKS-01, isolated in 2015 in China from a Holstein cow, is shown in black. **In (B) Maximum likelihood phylogenetic analysis of BCoV complete codes.** Strains in the tree are shown by name and colored according to their cluster as shown in (A). Clusters are indicated at the right side of the figure. Numbers at the branches of the tree show boostrap values. Only boostrap values higher than 90 are presented. Bar at the bottom of the tree denote distance.
